# Kinetics and isotype profile of antibody responses in rhesus macaques induced following vaccination with HPV 6, 11, 16 and 18 L1-virus-like particles formulated with or without Merck aluminum adjuvant

**DOI:** 10.1186/1476-8518-3-2

**Published:** 2005-04-20

**Authors:** Wanda Ruiz, William L McClements, Kathrin U Jansen, Mark T Esser

**Affiliations:** 1 Vaccine and Biologics Research Merck Research Laboratories 466 Devon Park Dr. Wayne, PA 19087-8630 USA; 2 Vaccine and Biologics Research Merck Research Laboratories West Point, PA 19486 USA

**Keywords:** human papillomavirus, vaccine, neutralizing antibody, Luminex

## Abstract

**Background:**

Human papillomaviruses (HPV) are the most common sexually transmitted viruses. Infection of the cervical epithelium by HPVs can lead to the development of cervical cancer. Recent advances in vaccine research have shown that immunization with papillomavirus-like particles (VLPs) containing the major structural viral protein, L1 from HPV 16 can provide protection from the establishment of a chronic HPV 16 infection and related cervical intraepithelial neoplasia (CIN) in baseline HPV 16 naïve women.

**Methods:**

To better understand the quantitative and qualitative effects of aluminum adjuvant on the immunogenic properties of an HPV 6, 11, 16 and 18L1 VLP vaccine, we used an HPV-specific, antibody isotyping assay and a competitive immunoassay that measures antibodies to neutralizing epitopes to profile sera from rhesus macaques immunized with the HPV L1 VLP vaccine formulated with or without aluminum adjuvant.

**Results:**

Immunization with VLPs formulated with the aluminum adjuvant elicited a significantly stronger immune response with higher peak antibody titers both at four weeks post vaccination (12.7 to 41.9-fold higher) as well as in the persistent phase at week 52 (4.3 to 26.7-fold higher) than that of VLPs alone. Furthermore, the aluminum adjuvant formulated HPV VLP vaccine elicited a predominantly T helper type 2 response, with high levels of IgG1 and IgG4 and low levels of IgG2. The vaccine also elicited high levels of serum IgA, which may be important in providing mucosal immunity to impart protection in the anogenital tract.

**Conclusion:**

These results show that the HPV 6, 11, 16 and 18 L1-VLP vaccine formulated with Merck aluminum adjuvant elicits a robust and durable immune response and holds promise as a vaccine for preventing cervical cancer.

## Background

Cervical cancer remains a leading cause of cancer-related deaths in women. HPV infection is the obligate first step in the development of cervical cancer [1]. Nearly a quarter of a million women die from cervical cancer and about half a million are diagnosed with this disease each year [2]. Cervical cancer accounts for 12% of all cancers in women and is the second most frequent gynecological malignancy in the world [3]. A large portion of this health burden is in the developing world, where women do not have access to good healthcare and Papanicolaou (Pap) screens. Although the widespread use of Pap screening in the developed world has increased the early detection of cervical dysplasia and cancer, thereby improving treatment outcomes for cervical cancer, it would be far more preferable to have a vaccine that blocks HPV infection, thereby preventing initiation of the disease process. Also, developing countries that usually do not have access to Pap screening and other preventive measures would further benefit from a vaccine that blocks HPV infection and its subsequent disease consequences. Therefore, there is a great need for an effective and generally well-tolerated HPV vaccine, having a low rate of occurrence of adverse events during administration.

Human papillomaviruses are small double-stranded DNA viruses. Infection with HPV is the most common viral sexually transmitted diseases worldwide [4]. HPV infects cutaneous and mucosal epithelial cells and causes benign and malignant hyperproliferative lesions, which includes genital warts and cervical cancer [5]. To date, more than 100 HPV types have been identified. Of these, 35 infect the genital tract [6,7]. Genital HPV types can be divided into two broad categories: low-risk types which cause genital warts, cervical dysplasia, but little cancer, and high-risk types, which cause dysplasia that can progress to cancer. HPV type 16 and 18 infection cause 70% of cancer cases and 25% of low grade cervical dysplasia [8]. HPV types 6 and 11, on the other hand, cause approximately 95% of genital warts (condylomata acuminata or venereal warts) and 25% of low grade cervical dysplasia (CIN1) [9,10]. Thus, a vaccine targeting HPV 6, 11, 16 and 18 will target the majority of HPV-related clinical disease.

Recently, we reported a proof-of-concept efficacy study for a prophylactic vaccine composed of HPV 16 L1 virus-like particles (VLP) formulated on Merck Aluminum Adjuvant (MAA) [11]. The double-blinded study randomized 2,391 women (16–23 years old) to receive either placebo or three doses of 40 μg of the HPV 16 VLPs in a 0, 2, and 6 month regimen. The primary efficacy endpoint was persistent HPV 16 infection, including HPV 16 related cervical dysplasia. Since the vaccine is being developed for prophylaxis against infection, the primary analysis was conducted in women who were naïve to HPV 16 at enrollment and remained free of HPV 16 infection through the completion of the vaccination series. Among placebo recipients within this cohort, 41 cases of persistent HPV 16 infection were detected. None of the women who received HPV 16 L1 VLP vaccine in this cohort developed an endpoint case [11].

Currently, we are investigating a quadrivalent vaccine, composed of HPV 6, 11, 16, and 18 L1 VLPs formulated with MAA. This vaccine has also been shown to be effective in preventing persistent infection and HPV 6, 11, 16 and 18 related cervical dysplasia [12]. To better understand the quantitative and qualitative effects of Merck aluminum adjuvant on the immunogenicity of the VLPs we used a novel HPV type-specific, antibody isotyping assay and a competitive Luminex immunoassay (cLIA) to measure HPV type-specific antibody responses in rhesus macaques. The results presented here show that formulation with Merck aluminum adjuvant increased the VLP's immunogenicity without affecting the isotype profile.

## Methods

### Vaccines

Virus-like particles were prepared as previously described (with modifications) from individual lysates of types- *Saccharomyces cerevisiae *expressing the L1 genes of HPV 6, 11, 16 and 18, respectively [13]. Equal concentrations of all four HPV VLPs were combined and used either directly, or adsorbed to MAA. A standard vaccine dose was composed of 2 μg each of the four VLP types, with or without 225 μg of MAA. The Merck Aluminum Adjuvant is a proprietary aluminum hydroxyphosphate sulfate based adjuvant used in other vaccines manufactured by Merck & Co., [14].

### Vaccine study on Rhesus Macaques

Groups of male and female Rhesus macaque (n = 5) were immunized at weeks 0, 8 and 24 with the two experimental vaccines described above. Serum was collected at weeks 0, 2, 4, 8, 10, 12, 16, 20, 24, 26, 28 and 52. The animals were maintained in accordance with the Institutional Animal Care and Use Committees of Merck Research Laboratories (West Point, PA).

### Antibodies

The monoclonal antibodies used in the HPV cLIA included H6.M48 [15] for HPV 6, K11.B2 for HPV 11, H16.V5 [16] for HPV 16 and H18.J4 [16] for HPV 18. These antibodies have been shown to be HPV type-specific and to bind to neutralizing epitopes [17]. The mAbs were conjugated to phycoerythrin (Chromaprobe, Aptos, CA) and used at a final concentration of 0.1 μg/mL each.

### HPV-specific immunoglobulin isotyping assay

A multiplexed antibody isotyping Luminex assay was developed to characterize the qualitative aspects of the HPV-specific humoral immune response. The multiplexed, isotyping assay was used to classify HPV-specific antibodies as IgM, IgA, IgE, IgG1, IgG2, IgG4, or total IgG. IgG3 was not evaluated since rhesus macaques do not make the IgG3 subclass of antibodies [18,19]. A panel of isotype-specific monoclonal antibodies to IgE, IgG1, IgG2, and IgG4 were purchased from either Sigma (St. Louis, MO) or Southern Biotechnology (Birmingham, AL) and isotype-specific polyclonal antibodies to IgA, IgM, and total IgG were purchased from Rockland Inc. (Gilbertsville, PA). These antibodies (Table 1) were evaluated for reactivity to human, rhesus and African green monkey immunoglobulins. Specificity of the antibodies to their respective immunoglobulin isotypes was established by a Luminex assay using purified human IgM, IgA, IgE, IgG1, IgG2, IgG3 and IgG4 covalently conjugated to Luminex microspheres. Microspheres were incubated overnight with Antibody Depleted Human Serum (ADHS), washed and detected using the isotype specific antibodies at the concentrations shown in Table 1. Optimum antibody concentrations, shown in Table 1, were established by comparing differing concentrations of the detection Abs in titrations of sera prior to immunization and positive sera from HPV 6, 11, 16 and 18L1 VLP vaccinated African green monkeys. Ab concentrations were chosen that gave the most sensitivity and the greatest signal to noise ratio between pre-immune and sera from vaccinated animals. Cross-reactivity between isotype detection antibodies was examined by conjugating purified human Igs to Luminex microspheres and incubating these microspheres with each of the isotyping antibodies at their chosen assay concentrations. In these experiments we detected some cross-reactivity of the anti IgG1 MAb towards IgG2 (71%) and the anti IgG2 towards IgG1 (58%). This cross-reactivity was the rationale for decreasing the concentration of the anti-IgG1 and IgG2 MAbs to 10 μg/mL (Table 1).

**Table 1:** Isotype specific antibodies and streptavidin-PE used in the HPV antibody isotyping assays

**Isotype Reagent**	**Type or MAb Identity**	**Specificity**	**Source**	**Supplier**	**Concentration**
*Primary*					
IgM	polyclonal	Monkey IgM (mu chain specific)	Goat	Rockland, Gilbertsville, PA	50 μg/mL
IgA	polyclonal	Monkey IgA (alpha chain specific)	Goat	Rockland, Gilbertsville, PA	25 μg/mL
IgE	HP-6029	Human IgE (epsilon chain specific)	Mouse	Southern Biotech, Birmingham, AL	25 μg/mL
IgG1	HP-6091or 8c/639	Human IgG1	Mouse	Sigma, St. Louis, MO	10 μg/mL
IgG2	HP-6014	Human IgG2	Mouse	Sigma, St. Louis, MO	10 μg/mL
IgG4	HP-6025	Human IgG4	Mouse	Sigma, St. Louis, MO	25 μg/mL
Total IgG	polyclonal	Monkey IgG (gamma chain specific)	Goat	Rockland, Gilbertsville, PA	5 μg/mL
*Secondary*					
PE- Streptavidin	NA	NA	NA	Rockland, Gilbertsville, PA	5 μg/mL

For the multiplexed, HPV-specific antibody isotyping assay, HPV 6, 11, 16, and 18 VLPs were covalently conjugated to four distinct Luminex microspheres identified as microsphere 6, 11, 16 and 18, respectively. Test sera from vaccinated rhesus macaques were serially diluted 5-fold starting from a dilution of 1:10 with ADHS and incubated overnight with the VLPs conjugated to microspheres (5,000 VLP-microspheres for each type in PBS + 1% Triton X-100 (PBST) in a total volume of 100 μl in a 1.2 μm hydrophilic, low protein binding, Durapore^® ^membrane filter plate (Millipore, Bedford, MA). The plate contents were washed 3 times with 200 μl of PBST and resuspended in PBST. HPV type-specific antibodies bound to the VLPs were incubated for 2 hours with biotinylated, isotype-specific secondary detection antibodies at the concentrations shown in Table 1. The microspheres were washed three times, and resuspended in PBST. The biotinylated antibodies were detected by incubation with streptavidin conjugated to phycoerythrin (Strep-PE) for 30 min at a final concentration of 5 μg/mL. The plates were washed 3 times and read on a Bio-plex analyzer purchased from Bio-Rad Laboratories, Inc. (Hercules, CA). The Bio-plex analyzer, based on Luminex xMAP technology, is a modified flow cytometer that allows for simultaneous quantitation of up to 100 analytes in a single well and reports Median Fluorescence Intensity (MFI) signals from the Strep-PE detection reagent [20]. End-point dilution titers were defined by comparing signals to those of ADHS, the negative control. For positivity, an MFI value had to be above the average of ADHS + 10 standard deviations and the end-point dilution had to demonstrate positivity at the previous lower dilution.

### HPV type-specific competitive Luminex Immunoassay (cLIA)

An optimized and previously validated HPV competitive Luminex Immunoassay (cLIA) first described by Opalka et al. [21] was used to quantify HPV 6, 11, 16, and 18 specific antibodies to known-neutralizing epitopes on VLPs. Using the same VLP-conjugated microspheres described above, antibody titers were determined using a competitive format, where HPV type-specific phycoerythrin (PE)-labeled monoclonal antibodies (MAbs) to known neutralizing epitopes compete with serum antibodies for binding to conformationally dependent, neutralizing epitopes. The fluorescent signals from bound HPV-specific MAbs are inversely proportional to the subject's neutralizing antibody titers. Relative inhibition of MAb binding in test serum was compared to a standard reference serum using a four-parameter logistic curve fit [22]. The reference sera for HPV 6, 11, 16 and 18 used for the standard curve were assigned arbitrary values expressed in milli-Merck Units per milliliter (mMU/mL). An antibody titer of >200 mMU/mL for HPV 11 has been shown to neutralize ~10^8 ^virions in the athymic mouse xenograft assay [23]. The titers for HPV 6 and 11 reference sera were previously determined in a pseudoneutralization assay [17]. The lower limit of quantitation for HPV6, 11 and 18 cLIAs is 8 mMU/mL and for HPV 16 is 12 mMU/mL.

### Statistical Analysis

Antibody titers obtained from the HPV cLIA were log-transformed and analyzed using a one-tailed, non-paired, Student's t-Test. P-values were obtained by comparing the average log-transformed titers of each of the 5 monkeys from week 2 to 52 within the two groups. The existence of a statistically significant difference between antibody titers from rhesus macaques vaccinated with VLP + MAA versus VLP alone was shown by a P-value of less than 0.05. Error bars represent the standard error of log-transformed titers.

## Results

### Vaccination with HPV VLPs formulated with aluminum adjuvant induces significantly higher titers than VLPs alone

**Figure 1:**
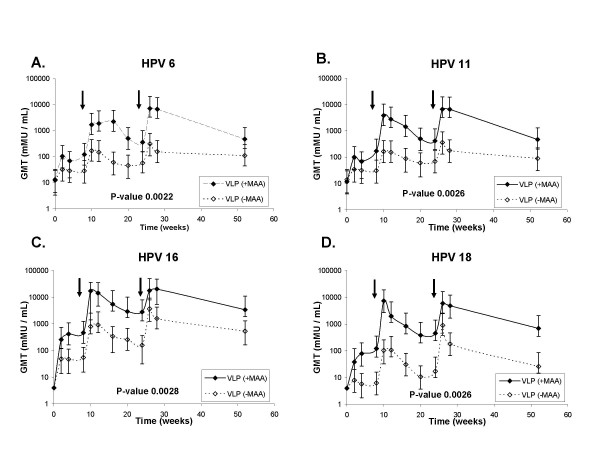
**HPV 6, 11, 16 and 18 type-specific antibody titers measured in the competitive Luminex Immunoassay (cLIA). **Sera from rhesus macaques immunized with 2 μg each of HPV 6, 11, 16 and 18L1-VLPs formulated with Merck Aluminum Adjuvant (MAA) (-◆-) or 2 μg each of HPV 6, 11, 16 and 18L1-VLPs alone (-◊ -) were collected a t the indicated time points and tested for Abs to neutralizing epitopes on HPV 6, 11, 16 and 18 using the HPV cLIA. Responses are reported as GMTs in milli-Merck Units per milliliter (mMU/mL) (n = 5 animals per group) for HPV 6, 11, 16 and 18 (Fig 1A, 1B. 1C. and 1D respectively). Arrows indicate vaccination boosts at weeks 8 and 24. A one-tailed, un-paired t-Test analysis was conducted on log-transformed antibody titers obtained from the cLIA. Error bars represent the standard error of the titers within each group.

The purpose of this study was to characterize the quantitative and qualitative effects of including MAA to the HPV 6, 11, 16 and 18 VLP vaccine. First we wanted to measure antibody titers to neutralizing epitopes on HPV L1 induced by a quadrivalent vaccine formulated with or without aluminum adjuvant using the HPV cLIA. Antibody titers for all four HPV types in both vaccine groups developed after the first vaccination, declined, and then increased again following each boost (Fig. 1). The data are consistent with a typical prime boost response. Peak antibody titers four weeks post dose 3, at week 28, for all four HPV types were 12.7 to 41.9-fold higher for the group vaccinated with VLPs + MAA compared to the group vaccinated with VLPs alone, and between 4.3 to 26.7-fold higher in the persistence phase at week 52 (Table 2A). Student's t-Tests of log-transformed antibody titers at week 52 of the VLP + MAA versus the VLP alone group for HPV 6, 11, 16 and 18 also revealed statistically significant P-values of 0.045, 0.026, 0.039 and 0.008 respectively. Student's t-Tests of log-transformed antibody titers from weeks 2–52 of the VLP+MAA versus the VLP alone group for HPV 6, 11, 16 and 18 also revealed statistically significant P-values of 0.0022, 0.0026, 0.0021, and 0.0026 respectively (Fig. 1).

**Table 2:** Fold difference in geometric mean titers of VLP+MAA-immunized group over VLP alone-immunized group.

**A.**	**HPV cLIA time (weeks)**											
**HPV type**	0	2	4	8	10	12	16	20	24	26	28	52
**HPV 6**	1.1	3.2	2.4	4.3	10.0	12.6	36.3	10.9	6.3	22.7	41.9	4.3
**HPV 11**	0.8	2.9	2.2	5.5	23.5	18.1	16.2	8.2	6.1	18.4	36.8	5.3
**HPV 16**	1.0	5.2	8.8	8.4	21.4	15.7	16.1	11.4	17.7	4.8	12.7	6.4
**HPV 18**	1.0	4.8	13.7	19.4	70.5	18.6	27.1	35.4	26.3	6.7	26.8	26.7

**B.**	**HPV Total IgG antibodies time (weeks)**											
**HPV type**	0	2	4	8	10	12	16	20	24	26	28	52

**HPV 6**	1.9	9.5	9.5	13.1	125.0	328.3	125.0	65.7	34.4	34.5	65.7	18.1
**HPV 11**	1.9	13.1	25.0	25.0	125.0	90.6	125.0	47.6	34.5	34.5	90.6	18.1
**HPV 16**	0.9	5.0	9.5	6.9	65.7	65.7	90.6	65.7	13.1	13.1	65.7	25.0
**HPV 18**	0.7	9.5	13.1	25.0	125.0	47.6	65.7	65.7	25.0	47.6	125.0	90.6

**Figure 2:**
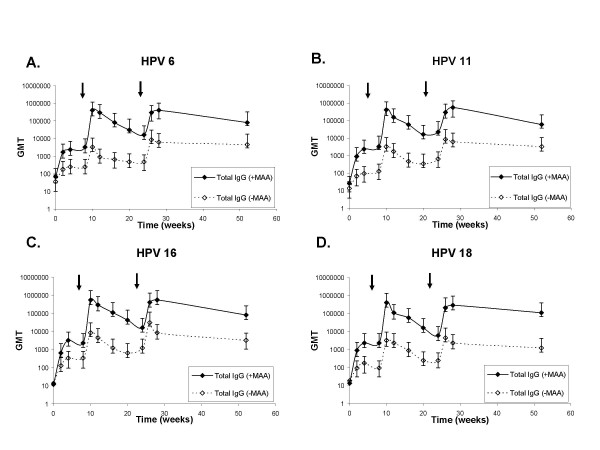
**HPV 6, 11, 16 and 18 L1 VLP-specific total IgG antibody titers. **Sera from rhesus macaques immunized at week 0, 8 and 24 with 2 μg each of HPV 6, 11, 16 and 18L1-VLP formulated with Merck Aluminum Adjuvant (+MAA) (-◆-) or 2 μg each of HPV 6, 11, 16 and 18L1-VLPs alone (-MAA) (-◊ -) were collected at the indicated time points and tested for HPV 6, 11, 16 and 18 L1 VLP specific total IgG (-◆-, -◊ -) titers in a multiplexed detection assay. Responses are reported as the geometric means of end point dilution titers (n = 5 animals per group) for HPV 6, 11, 16 and 18 (2A, B, C, and D respectively). Arrows indicate vaccination boosts at weeks 8 and 24. The starting dilution for each sample was 1:10 and responses above an end point dilution of 10 were considered positive.

We also wanted to measure the total HPV-specific IgG levels to determine whether the formulation with MAA increased the total HPV-specific Ab titers. The total HPV-specific IgG peak titers, and titers seven months post dose three were consistently higher in the VLP+MAA group compared to the VLP group alone (Fig. 2). An anamnestic response was observed after each vaccine boost and total IgG levels were detectable through one year. The peak antibody titers four weeks post dose 3, at week 28, in the VLP+MAA group were between 65.7 to 125.0-fold higher than those in the VLP alone group throughout the vaccination series and between 18.1 to 90.6-fold higher in the persistence phase, at week 52 (Table 2B).

### HPV-specific antibody isotype responses

**Figure 3:**
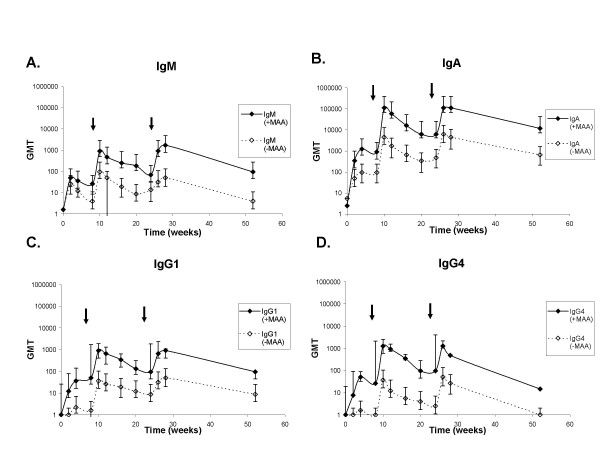
**HPV L1-VLP specific IgM, IgA, IgG1 and IgG4 antibody titers. **Sera from rhesus macaques immunized at week 0, 8 and 24 with 2 μg each of HPV 6, 11, 16 and 18 L1-VLPs formulated with Merck Aluminum Adjuvant (+MAA) (-◆-) or 2 μg each of HPV 6, 11, 16 and 18L1-VLPs alone (-MAA) (-◊ -) were collected at the indicated time points and tested for HPV L1 VLP specific (A) IgM, (B) IgA, (C) IgG1 and (D) IgG4 titers in a multiplexed detection assay. Responses are reported as GMTs (n = 5 monkeys per group) for HPV 16. Arrows indicate vaccination boosts at weeks 8 and 24. The starting dilution for each sample was 1:10 and responses above an end point dilution of 10 were considered above background. Graphs shown are for HPV 16 and are representative of HPV 6, 11, and 18.

To characterize the antibody isotypes elicited by VLPs formulated with MAA or VLPs, alone we modified a multiplexed Ab isotyping assay first developed to measure Ab isotypes in humans [24]. These changes included using detection mAbs that react with human, rhesus and African green monkey Igs, where possible (Table 1). In addition we used biotinylated Abs so that we could use a common streptavidin-PE detection reagent. This novel HPV-specific antibody isotyping assay was found to be specific and sensitive for the detection of rhesus macaque IgM, IgA, IgE, IgG4 and total IgG. Low cross reactivity was seen with the anti-IgG1 and IgG2 antibodies to purified human IgG2 and IgG1 respectively. Responses to these antibody isotypes were measured separately for HPV 6, 11, 16 and 18 and were similar to each other. Results for HPV16 responses which are representative of all four types are shown in figure 3.

We first examined the HPV-specific IgM responses because IgM antibodies are the first to be produced following vaccination and would indicate a primary immune response to the vaccine. As expected, an IgM response for all four types was measured after the first immunization at week 0 and is shown for the representative type HPV 16 (Fig. 3A). Interestingly, two additional primary IgM responses were observed after each of the booster vaccinations given at weeks 8 and 24 in both the VLP and VLP+MAA groups of the study (Fig. 3A). IgM responses in the VLP alone group returned to baseline by week 52, which was seven months post dose 3, while an IgM antibody response in the VLP + MAA group was detectable at week 52.

Since HPVs are sexually transmitted viruses that cross the mucosal barrier in the anogenital tract, we next examined whether the VLPs induced an IgA response and what effect formulation with MAA would have on an IgA response. In theory, the induction of IgA would greatly enhance the effectiveness of an HPV vaccine. Both vaccine groups demonstrated type-specific IgA responses to the VLPs that were maintained through one year (Fig. 3B). Each vaccine boost elicited secondary IgA responses in both vaccination study groups (Fig. 3B). Higher IgA end-point dilution titers were observed in the rhesus macaques vaccinated with VLP + MAA compared to those vaccinated with VLP alone.

We also examined whether there was an IgE response induced following vaccination. The geometric mean titers (GMTs) of IgE responses for the two vaccine groups were not above background, however one animal in the VLP group had a detectable IgE response at week 4 for HPV 6 and 16, and at week 24 for HPV 16 (data not shown). This response was just barely above the limit of detection and was not detected following the booster doses given at weeks 8 and 24.

We next measured the IgG subtypes to determine whether formulation with Merck aluminum adjuvant affected the Ig isotype profile. Characterizing the IgG isotype profile would also provide insight into whether formulation with MAA altered the TH1 or TH2 profile of the immune response. The main IgG subclass observed in both vaccination groups was IgG1 (Fig. 3C). Similar to responses observed for total IgG, secondary IgG1 responses were observed after each boost, and titers were maintained through one year in the VLP + MAA group (Fig. 3C). However, the group vaccinated with VLP alone had detectable titers only after the second immunization and these titers approached baseline values at one year (Fig 3C). We also observed high levels of IgG4 and Ab titers in the VLP + MAA group were detected early after each vaccination, but were undetectable by week 52 (Fig. 3D). The monkeys vaccinated with VLPs alone only demonstrated IgG4 responses after each vaccination boost, but the titers fell below the limit of detection soon after (Fig. 3D). We did not detect a strong IgG2 response in the VLP-alone group and IgG2 levels in the VLP + MAA group were observed only immediately following vaccination (data not shown). However, because we observed some cross-reactivity of the anti-IgG2 MAb to human IgG1, we cannot rule out the possibility that the IgG2 signals observed were due to cross-reactivity of the anti-IgG2 MAb to the high levels of rhesus IgG1 Ab (Fig. 3A). HPV-specific IgG3 responses were not measured because rhesus macaques do not make the IgG3 subclass of antibodies [18,19]. In summary, higher total IgG, IgG1, and IgG4 Ab titers were observed in the rhesus macaques vaccinated with VLP + MAA compared to those vaccinated with VLPs alone.

## Discussion

This is the first study to directly examine the effects of the Merck aluminum adjuvant on immune responses to a quadrivalent HPV 6, 11, 16 and 18 L1 vaccine. The results here were generated using a novel HPV-type specific antibody isotyping assay to characterize the immunoglobulin subclasses to HPV 6, 11, 16 and 18 in rhesus macaques, and a competitive Luminex immunoassay (cLIA) that measures HPV-type specific antibodies to known neutralizing epitopes. The quadrivalent HPV L1 VLP vaccine formulated with MAA induced higher titers for total IgG (Fig. 2), as well as for all Ig isotypes measured (Fig. 3) compared to the vaccine without MAA. Also the MAA-formulated vaccine induced higher overall antibody titers to neutralizing epitopes compared to one without MAA (Fig. 1). Strong immune responses were elicited against all four HPV types and high antibody titers persisted through one year.

Results from the HPV cLIA showed that the vaccine induced antibodies that competed with MAbs to known neutralizing epitopes, and are, thus, theoretically able to neutralize HPV. It is worth noting that we have previously shown that a competitive radioimmunoassay (cRIA) [25] and the cLIA correlate well with an HPV 11 neutralization assay. Antibody titers in the cLIA also followed a "prime-boost" response wherein antibody titers were higher after every vaccine dose. Furthermore, cLIA titers show that formulation of the vaccine with MAA significantly increased peak neutralizing titers, and, more importantly, the differences in the responses were durable, persisting through one year.

Previously, we reported that the Merck HPV 16 L1 VLP vaccine induced a predominantly TH2 immune response with IL-4-producing T-helper cells, high levels of neutralizing Abs and low levels of IFN-γ secreting CD8^+ ^or CD4^+ ^cells [26]. Given that the HPV quadrivalent vaccine also contains the L1 capsid proteins, the response was also expected to be TH2-like [27]. Using the HPV isotyping assay, which is a direct binding assay detecting antibodies to conserved epitopes shared across multiple HPV types, we showed that the isotype profile of the vaccinated rhesus macaques was consistent with a TH2-like immune response. Interestingly, the formulation with MAA did not affect the overall isotype profile of the vaccinated monkeys.

Predictably, abundant amounts of IgG antibody isotype were found, and of all the IgG subclasses, IgG1 was most readily detected. The abundant presence of IgG1 elicited in both vaccination groups affirms that the vaccines did induce immune responses through a TH2 pathway. It is also interesting to note that the next abundant IgG subclass detected, primarily detected in the VLP+MAA group, was IgG4 (Fig. 3D). IgG4 is more characteristic of a TH1 response and, by inducing this IgG subclass, the vaccine is shown to be also effective in eliciting a cellular (TH1) immune response, albeit, predictably not long lasting. It is worth noting that we previously observed low levels of IFN-γ secreting CD8^+ ^and CD4^+ ^T-cells [26], suggesting the vaccine induces a low level cellular immune response. Similar to what we observed in rhesus macaques, isotype analysis of sera from women vaccinated with the HPV 11 VLP vaccine formulated with MAA also detected little to no levels of IgG2 [24]. Isotype analysis performed on an epidemiological study by Wang et al. also found no IgG2 antibody, which they attributed to the unfavorable cytokine profile of HPV infection for the induction of IgG2 [28]. Detection of the IgG3 subclass was not performed, because rhesus macaques do not make IgG3 [18,19].

We also measured high levels of serum IgA observed in vaccinated animals. IgA, the secretory immunoglobulin, is thought to be very important in blocking viral entry into mucosal tissues [29]. These findings are consistent with what we have observe in the sera from women vaccinated with the HPV 11 VLP vaccine [24]. The elicitation of IgA should be advantageous for a vaccine that prevents HPV infection in defending against entry of HPV, which commonly enters through the urogenital tract. The high levels of IgA elicited by the vaccine may also be one reason why the vaccine has been efficacious in human clinical trials [11]. IgE responses were measured because IgE antibodies are implicated in allergic symptoms. Consistent IgE antibody responses were not observed in either group. Only one animal in the VLP alone vaccine group had detectable IgE, but the response was barely above the limit of detection and transient as it was not detected following the booster immunizations. This is consistent with the observation of no allergic reactions or severe adverse events in the monkeys following vaccination. These observations are consistent with our clinical findings that a VLP + MAA vaccine was safe and well-tolerated [30]. In summary, the isotype analysis data are consistent with the VLP+MAA vaccine inducing primarily a TH2-like immune response with high titers of IgA and IgG1 antibodies.

In these isotyping studies, we used polyclonal goat anti-monkey IgM, IgA, and total IgG; because monkey-specific reagents are not available for the IgG subtypes and IgE, we used anti-human mouse MAbs to measure IgG1, IgG2, IgG4, and IgE responses. The polyclonal anti-IgA, IgM and total IgG reagents were selected for their specificity and their ability to broadly detect African Green monkey, rhesus macaque and human antibody isotypes. The various differences between human, rhesus macaque and African green monkey sera could account for changes in the sensitivity and specificity of the antibody-isotyping reagents and so the appropriate antibody reagent concentrations used in the HPV type-specific antibody isotyping assay were optimized for use with rhesus macaque serum.

Due to the unavailability of purified rhesus Igs, we used purified human Igs to determine the specificities of the type-specific antibody isotyping reagents. There was some observed reactivity of the anti- human IgG2 antibody to purified human IgG1, and vice versa, but these data were obtained using purified human Igs and the differences between human and rhesus antibodies may account for why we saw a strong IgG1 response with low IgG2 titers (Fig. 3). However, because we cannot rule out that the IgG2 titers were merely a reflection of the cross-reactivity to the high IgG1 titers, we cannot definitively say that there were any IgG2 responses observed.

In some cases, the test animals had low Ab titers to the VLPs prior to vaccination. This was not unexpected since more than 100 papillomaviruses have been identified [31] and many of these share conserved structural epitopes that would cross react in a direct binding assay. For this reason, we used Antibody Depleted Human Serum (ADHS) as a negative control rather than sera from rhesus macaques prior to vaccination.

Currently, the only FDA approved adjuvants for use with prophylactic vaccines are aluminum based [32]. The purpose of this study was to evaluate the effect of the Merck Aluminum Adjuvant on the immunogenicity of the HPV L1 VLP 6, 11, 16 and 18 vaccine and our results show that the addition of the MAA significantly increases the immunogenicity of the vaccine. In sharp contrast to our results, Harro et al. have reported that formulation with adjuvant did not significantly enhance the immunogenicity of an HPV VLP vaccine [33]. In that study, 72 human volunteers were randomized to receive placebo or an HPV 16 L1 VLP vaccine produced in insect cells. Vaccine recipients were given a dose of 10 μg or 50 μg of VLPs formulated without adjuvant, with an aluminum adjuvant, or with MF59 adjuvant. Since no significant differences in neutralizing antibody titers and ELISA titers were found in subjects in all three groups, the authors concluded adjuvant was not required. Our results were obtained using the quadrivalent Merck HPV-L1 VLP vaccine, which is formulated with Merck aluminum adjuvant. While the mechanisms by which aluminum adjuvants enhance immunogenicity are poorly understood, they may act to form an antigen depot, allowing the vaccine to linger at the site of administration and perhaps, more importantly, stimulate the immune system by inducing higher neutralizing Ab titers [34]. The inclusion of MAA to the vaccine did not induce a qualitatively different humoral immune response in that the isotype profile was similar between animals vaccinated with the two quadrivalent vaccines (Fig. 3). However, data from both the antibody isotyping assays and the cLIA showed that the inclusion of MAA to the VLP vaccine induced significantly higher Ab titers (Fig. 1, 2, 3). These data clearly show a benefit to the formulation of the quadrivalent HPV-L1 VLPs with MAA.

The differences observed in the immune responses elicited by the vaccine reported by Harro et al. and the Merck quadrivalent HPV vaccine may be attributed to several factors. First, would be the differences in the formulation and purification processes of the vaccines. The vaccine reported by Harro et al. was produced in insect cells as opposed to yeast for the Merck vaccine. Second, the differences in vaccine recipients, humans and rhesus macaques may respond differently to adjuvanted HPV VLP vaccines. Lastly, differences in formulation and composition of the two aluminum adjuvants used could have profound impacts on immunogenicity. Different aluminum salt adjuvants were used in these two studies; our study adsorbed VLPs to a proprietary formulation of aluminum hydroxyphosphate sulfate (MAA), while the Harro et al. study used aluminum potassium sulfate. Without a head to head comparison of the two vaccines, it is difficult to establish why the results are different. However, the magnitude and durability of antibody responses found in the quadrivalent VLP+MAA vaccinated rhesus macaques reported in this study are consistent with those found in human clinical trials of the HPV 11 or HPV 16 monovalent vaccines [30].

The isotyping and cLIA assays described in this study are useful in characterizing the immune responses to HPV virions after natural infection or following vaccination. The novel cLIA utilized in this study is a robust and sensitive method for detecting HPV-specific antibodies in sera to known neutralizing epitopes on HPV virions and has proven to be a valuable tool for monitoring HPV immune responses in human clinical trials of an HPV 6, 11, 16 and 18L1-VLP vaccine. These assays have potential use in future epidemiology studies and other vaccine clinical trials.

## Conclusion

The results presented here show that an HPV 6, 11, 16 and 18 L1-VLP vaccine formulated with Merck aluminum adjuvant has increased immunogenicity without affecting the isotype profile. The VLPs formulated with Merck aluminum adjuvant elicited a robust and durable immune response that lasted up to 52 weeks. This vaccine holds promise as a vaccine for preventing cervical cancer.

## List of abbreviations

MAb, monoclonal antibody; PBS, phosphate-buffered saline; cLIA, competitive Luminex immunoassay; ADHS, antibody depleted human serum; VLP, virus-like particles; HPV, human papillomavirus; GMT, geometric mean titers; Ig, immunoglobulin; Ab, antibody.

## Competing interests

W. Ruiz, W. McClements, K. Jansen and M. Esser were all employees of Merck Research Laboratories, a division of Merck & Co., Inc. when this study was performed and potentially own stock and/or hold stock options in the Company. Merck is developing a quadrivalent HPV vaccine. Merck also funded this study in its entirety.

## Authors' contributions

W. McClements and K. Jansen designed the immunization study. M. Esser and W. Ruiz developed the competitive Luminex immunoassay and the new isotyping assays. W. Ruiz performed all the laboratory assays. W. Ruiz and M. Esser wrote the manuscript and all authors read and approved the final manuscript.
